# M2 Macrophages Infiltrating Epithelial Ovarian Cancer Express MDR1: A Feature That May Account for the Poor Prognosis

**DOI:** 10.3390/cells9051224

**Published:** 2020-05-15

**Authors:** Susann Badmann, Sabine Heublein, Doris Mayr, Anna Reischer, Yue Liao, Thomas Kolben, Susanne Beyer, Anna Hester, Christine Zeder-Goess, Alexander Burges, Sven Mahner, Udo Jeschke, Fabian Trillsch, Bastian Czogalla

**Affiliations:** 1 Department of Obstetrics and Gynecology, University Hospital, LMU Munich, Marchioninistraße 15, 81377 Munich, Germany; Susann.Badmann@med.uni-muenchen.de (S.B.); Sabine.Heublein@med.uni-heidelberg.de (S.H.); Yue.Liao@med.uni-muenchen.de (Y.L.); Thomas.Kolben@med.uni-muenchen.de (T.K.); Susanne.Beyer@med.uni-muenchen.de (S.B.); Anna.Hester@med.uni-muenchen.de (A.H.); Christine.Zeder-Goess@med.uni-muenchen.de (C.Z.-G.); Alexander.Burges@med.uni-muenchen.de (A.B.); Sven.Mahner@med.uni-muenchen.de (S.M.); Udo.Jeschke@med.uni-muenchen.de (U.J.); Fabian.Trillsch@med.uni-muenchen.de (F.T.); 2 Department of Obstetrics and Gynecology, Heidelberg University Hospital, Ruprecht-Karls-University of Heidelberg, Im Neuenheimer Feld 440, 69120 Heidelberg, Germany; 3 Institute of Pathology, Faculty of Medicine, LMU Munich, Thalkirchner Straße 36, 80337 Munich, Germany; doris.mayr@med.uni-muenchen.de; 4 Department of Medicine III, University Hospital, LMU Munich, Marchioninistraße 15, 81377 Munich, Germany; Anna.Reischer@med.uni-muenchen.de; 5 Department of Obstretrics and Gynecology, University Hospital Augsburg, Stenglinstraße 2, 86156 Augsburg, Germany

**Keywords:** MDR1, M2 Macrophages, ovarian cancer, TA-MUC1, prognosis

## Abstract

Multi drug resistance protein 1 (MDR1) expression on tumor cells has been widely investigated in context of drug resistance. However, the role of MDR1 on the immune cell infiltrate of solid tumors remains unknown. The aim of this study was to analyze the prognostic significance of a MDR1+ immune cell infiltrate in epithelial ovarian cancer (EOC) and to identify the MDR1+ leucocyte subpopulation. MDR1 expression was analyzed by immunohistochemistry in 156 EOC samples. In addition to MDR1+ cancer cells, we detected a MDR1+ leucocyte infiltrate (high infiltrate >4 leucocytes per field of view). Correlations and survival analyses were calculated. To identify immune cell subpopulations immunofluorescence double staining was performed. The MDR1+ leucocyte infiltrate was associated with human epidermal growth factor receptor 2 (HER2) (cc = 0.258, *p* = 0.005) and tumor-associated mucin 1 (TA-MUC1) (cc = 0.202, *p* = 0.022) expression on cancer cells. A high MDR1+ leucocyte infiltrate was associated with impaired survival, especially in patients whose carcinoma showed either serous histology (median OS 28.80 vs. 50.64 months, *p* = 0.027, n = 91) or TA-MUC1 expression (median OS 30.60 vs. 63.36 months, *p* = 0.015, n = 110). Similar findings for PFS suggest an influence of MDR1+ immune cells on the development of chemoresistance. A Cox regression analysis confirmed the independency of a high MDR1+ leucocyte infiltrate as prognostic factor. M2 macrophages were identified as main part of the MDR1+ leucocyte infiltrate expressing MDR1 as well as the M2 marker CD163 and the pan-macrophage marker CD68. Infiltration of MDR1+ leucocytes, mostly M2 macrophages, is associated with poor prognosis of EOC patients. Further understanding of the interaction of M2 macrophages, MDR1 and TA-MUC1 appears to be a key aspect to overcome chemoresistance in ovarian cancer.

## 1. Introduction

Ovarian cancer is one of the most lethal tumor entities [1]. Histologically epithelial ovarian cancer (EOC) is classified into five main subtypes: high-grade serous, low-grade serous, mucinous, endometrioid, and clear-cell histology [2]. Especially low- and high-grade serous ovarian carcinoma cause approximately 70% of EOC [3]. According to guidelines from the European Society of Gynecologic Oncology and European Society for Medical Oncology, EOC is treated by initial tumor debulking surgery followed by adjuvant platinum-based chemotherapy combined with anti-angiogenic agents or followed by poly-ADP-ribose-polymerase inhibitors [4]. However, frequent development of chemoresistance is one of the essential problems limiting sufficient treatment of ovarian cancer. To overcome this issue, a better understanding of molecular alterations in ovarian cancer is needed. Szakacs et al. described three major mechanisms of multidrug resistance (MDR): reduced uptake of hydrophilic drugs, changes in tumor cells to circumvent the cytotoxic effect, and increased removal of hydrophobic drugs by transporters of the ATP-binding cassette family [5]. Multi drug resistance protein 1 (MDR1) is one of those ATP dependent efflux pumps, which is expressed in many human tissues to protect the cells from toxins [6]. In various cancer types, including ovarian cancer, induction of MDR1 expression correlates with decreased therapy response and overall survival (OS) [7,8]. Therefore, the contribution of MDR1 expression on tumor cells to chemoresistance has been studied and specific inhibitors have been tested in clinical trials, so far with modest benefit [5,7,8].

Over time, it has been realized that the mechanisms of drug resistance can only be explained partly by intrinsic properties of ovarian cancer cells. An increased number of studies demonstrated the importance of the tumor microenvironment (TME) in cancer progression, angiogenesis, dissemination, and chemoresistance. Its composition of extracellular matrix, secreted factors and stromal cells is very variable. Crosstalk between cancer and stromal cells often leads to a pro-tumoral TME [9]. Especially macrophages are attributed to an immunosuppressive effect. They play a prominent active role from early carcinogenesis to tumor progression including metastasis [10]. In response to environmental stimuli macrophages polarize either to pro-inflammatory (M1) or anti-inflammatory (M2) phenotypes [11]. In metastatic and solid tumors, macrophages show mainly the pro-tumoral M2-like phenotype and facilitate cancer growth, cell migration, and angiogenesis by producing a large number of growth factors, extracellular matrix remodeling molecules, and cytokines [11,12].

Indeed, MDR1 expression on ovarian cancer cells is a critical driver of worse prognosis and therapy resistance. Recently several studies reported its expression on immune cells in different extend with functional consequences on their migration, differentiation or survival reviewed in Bossennec et al. [13]. In context of tumor treatment MDR1 expression rescues leucocytes from cytotoxic agents. This effect would be desirable in case of antitumoral immune cell subpopulations. But often protumoral subpopulations like M2 macrophages and Tregs are found in the tumor environment. If protumoral immune cell subpopulations express MDR1 to a higher extend, they would gain a survival advantages trough the treatment leading to worse prognosis and therapy response. The aim of this study is to identify a possible prognostic impact of MDR1+ infiltrating immune cells in EOC and to identify the MDR1+ leucocyte subpopulation for a better understanding of their contribution to chemoresistance.

## 2. Materials and Methods

### 2.1. Patients and Specimens

In this study, formalin-fixated and paraffin-embedded (FFPE) tissue samples of 156 patients who underwent debulking surgery for EOC from 1990 to 2002 at the Department of Obstetrics and Gynecology, Ludwig-Maximilian-University Munich, were analyzed. Only patients with pathologically confirmed EOC were included. Exclusion criteria were neoadjuvant chemotherapy as well as benign or borderline tumors. Previously, other pathological parameters were investigated in the same patients’ collective, which enables correlation analysis [14,15,16,17]. Clinical data derived from patients’ charts, follow up data from Munich Cancer Registry. Histological subtype and grading were determined by specialists at the Department of Pathology, Ludwig-Maximilian-University Munich. Staging was performed according to the WHO and FIGO classification (2014). The clinicopathologic characteristics of the analyzed ovarian cancer patients are listed in Table 1. Information to FIGO stage and grading are missing in 5 respectively 9 cases. Unfortunately, information on the residual disease after primary surgery is only available in 13 cases.

### 2.2. Ethical Approval

This study was approved by the Ethics Committee of the Ludwig-Maximilian-University Munich (approval number 227-09, 18-392, and 19-972). The ovarian cancer specimens were obtained in clinically indicated surgeries and initially used for histopathological diagnostics. When the current study was performed, all diagnostic procedures were completed, and the patients’ data were anonymized. The ethical principles adopted in the Declaration of Helsinki 1975 have been respected.

### 2.3. Immunohistochemistry

For immunohistochemistry staining, a protocol previously described by our lab was used [17]. Tissue micro arrays of FFPE EOC samples were deparaffinized for 20 min in roticlear (Roth, Kalsruhe, Germany), incubated for 20 min in methanol containing 3% $H_{2}O_{2}$ to quench the endogen peroxidase, rehydrated in a descending series of alcohol (100–50%), and cooked for 5 min in a pressure cooker containing a sodium-citrate buffer (0.1 M citric acid, 0.1 M sodium citrate, pH = 6.0). After cooling down, the slides were washed in distilled water and phosphate buffered saline (PBS), blocked and incubated with the anti-MDR1 primary antibody (monoclonal rabbit IgG; Abcam, Cambridge, UK) in a 1:100 dilution at 4 °C overnight (16 h). For detection a polymer system bound with secondary antibodies anti-mouse/rabbit and horse radish peroxidase (ZytoChem Plus HRP Polymer System mouse/rabbit; Zytomed, Berlin, Germany) was applied for 30 min at room temperature and for visualization the chromogen substrate solution containing 3,3-diamino-benzidine (Dako, Carpinteria, CA, USA) was added. Counterstaining was performed with Mayer’s acidic haemalum (Waldeck, Münster, Germany). Tissue from human small intestine, kidney, liver, tonsil and fallopian tube served as system control (Supplementary file Figure S1). As additional control, MDR1 staining of healthy ovarian tissue was performed (Supplementary file Figure S2). The intensity of MDR1 expression on tumor cells and the percentage of MDR1+ tumor cells was assessed in a semi-quantitative manner using the immunoreactive score (IRS) [18]. The MDR1+ leucocyte infiltrate (intratumoral and in the peritumoral stroma) was quantified by counting positive stained leucocytes per field of view (25× lens) and grouped by low infiltrate (≤4 leucocytes per field of view) and high infiltrate (>4). Mean values of infiltrating leucocytes detected in three spots of the same individual were calculated.

### 2.4. Immunofluorescence

To clearly distinguish between tumor and infiltrating immune cells and to characterize the immune cell subpopulation immunofluorescence double staining for MDR1 and CD45 as common leucocyte marker and other accepted markers for leucocyte subpopulations (CD3, CD56, CD68, CD163, TLR2) was performed. The slides were pre-treated like for immunohistochemistry. To prevent unspecific binding of the primary antibody a blocking solution (UltraVision Protein Block; Thermo Scientific, Lab Vision, Fremont, CA, USA) was applied to the slides for 15 min. The slides were incubated for 16 h with a mixed solution of the primary antibodies (Supplementary file Table S1). After washing the slides two times with PBS, fluorophore-labeled secondary antibodies were applied for 30 min in the dark at room temperature (Supplementary file Table S1). Finally, the slides were covered with mounting medium (Vectashield H-1200; Vector Laboratories, Burlingame, CA, USA) containing DAPI for nuclear counterstaining. All double staining were observed in 20×, 40× and 63× magnification using a confocal laser microscope (Axiophot fluorescent microscope; Zeiss, Oberkochen, Germany) and analyzed with the corresponding software AxioVision. The immune cell subpopulations were quantified by counting positive stained cells for CD68, CD3 and CD56 per field of view (20× lens, n = 12 each).

### 2.5. Statistical Analysis

SPSS 25.0 (IBM Corporation, Armonk, NY, USA) was used for data processing and statistical analysis of this study. To compare the distribution of more than two independent samples, like histological subtype, Kruskal-Wallis H-test was used [19]. Bivariate correlations between clinical and pathological data have been calculated with Spearman’s analysis [20]. Survival times of different subgroups were analyzed by log-rank testing and displayed in Kaplan-Meier curves [21]. For cut-off point selection ROC analyses were performed and the Youden index (sensitivity + specificity − 1) was maximized [22,23]. Cox regression models [24] were applied for multivariate analysis. *p*-values ≤0.05 were considered as statistically significant.

## 3. Results

### 3.1. MDR1 Expression on Ovarian Cancer Cells and on Infiltrating Leucocytes

**Figure 1:**
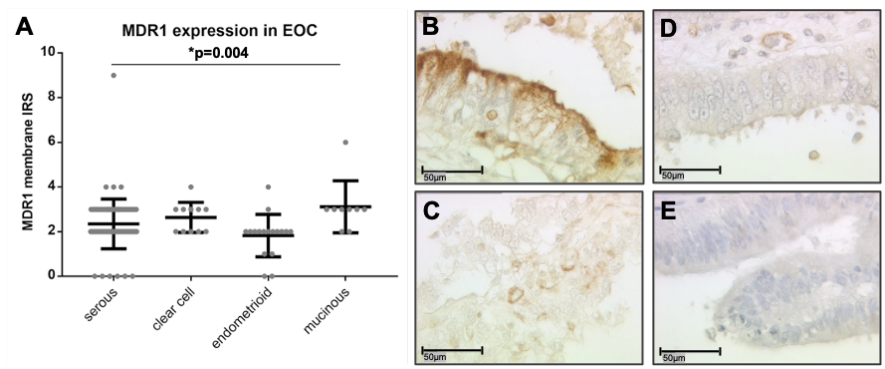
MDR1 immunostaining of ovarian cancer cells. Membranous expression of MDR1 on ovarian cancer cells differs between the subtypes (**A**, *p* = 0.004) with mucinous (**B**, IRS = 6) and clear cell (**C**, IRS = 4) showing a higher expression than serous (**D**, IRS = 3) and endometrioid (**E**, IRS = 2). (**B**–**E**) are shown in 40× magnification (scale bar = 50 µm), 25× magnification is provided in the Supplementary Figure S3.

The clinicopathologic characteristics of the analyzed ovarian cancer patients are listed in Table 1. Out of the 156 stained ovarian cancer specimens, 131 (84%) showed positive MDR1 expression on tumor cells (IRS > 0). In contrast to the area of the tumor, healthy ovarian tissue was not positive for MDR1 (Supplementary file Figure S2). The median IRS for membranous staining of the tumor cells was 2 (total range 0–9). Differences in membranous MDR1 expression between the histological subtypes were detected (*p* = 0.004). Clear cell and mucinous carcinomas tended to have higher MDR1 expression levels (median IRS = 3) than serous and endometrioid tumors (median IRS = 2) (Figure 1A–E).

**Figure 2:**
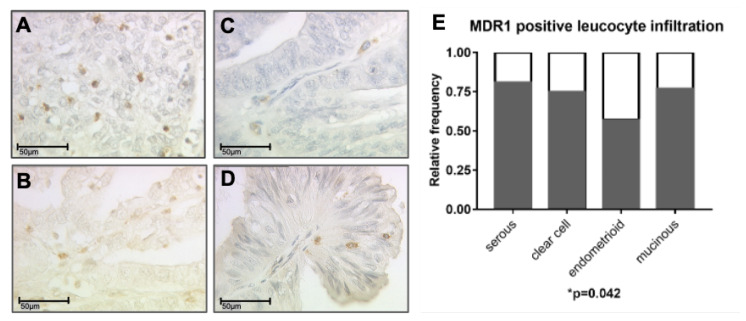
A MDR1+ leucocyte infiltrate was detected by immunohistochemistry in all subtypes: serous (**A**), clear cell (**B**), endometrioid (**C**) and mucinous carcinoma (**D**). (**A**–**D**) are shown in 40× magnification (scale bar = 50 µm), 25× magnification is provided in the supplementary Figure S4. The highest relative frequency of cases with MDR1+ leucocyte infiltration was found for serous histology (E, *p* = 0.042) followed by mucinous, clear cell and endometrioid.

In 120 (76.9%) of 156 cases a MDR1+ immune cell infiltrate (>0 per field of view) to the tumor and peritumoral stroma was detected (Figure 2A–D). 74 cases (47.4%) showed a high infiltration (>4 per field of view), whereas in 36 cases (23.1%) no infiltrate was observed. In case of MDR1 expression on tumor cells (IRS > 0, n = 131), 114 patients (87%) showed also a MDR1+ immune cell infiltrate, 73 patients (55.7%) showed a high MDR1+ leucocyte infiltration. To clearly distinguish between tumor and infiltrating immune cells immunofluorescence double staining for MDR1 and CD45 as common pan-leucocyte marker was performed (shown in the Supplementary file Figure S7). In healthy ovarian tissue only sporadic MDR1+ infiltrating immune cells were found, but in lower density compared to EOC (Supplementary file Figure S2). There was no significant difference in the extent of MDR1+ leucocyte infiltration between the histological subtypes, but in serous subtype the relative frequency of patients showing a MDR1+ immune cell infiltrate was highest followed by patients with mucinous, clear cell and endometrioid histology (*p* = 0.042) (Figure 2E). The relative frequency was calculated for each subtype as ratio of number of patients showing a MDR1+ leucocyte infiltrate (>0 per field of view) and total number of patients analyzed.

### 3.2. MDR1+ Leucocyte Infiltration Correlates with Tumor Specific Antigens HER2 and TA-MUC1

Correlation analyses between the MDR1+ leucocyte infiltrate (number of MDR1+ leucocytes per field of view) and clinicopathological data (age, subtype, FIGO, primary tumor expansion (pT), nodal status (pN), and grading) and other prognostic parameters of the study cohort, that had been identified previously by our group, were conducted for the whole collective. Significant correlations of the MDR1+ leucocyte infiltrate with the tumor specific antigens human epidermal growth factor receptor 2 (HER2) (cc = 0.258, *p* = 0.005) and tumor-associated mucin 1 (TA-MUC1) (cc = 0.202, *p* = 0.022) were found (Table 2), but no further correlations with the above mentioned clinicopathological parameters (Supplementary file Table S2).

### 3.3. High MDR1+ Immune Cell Infiltration Is Associated With Worse Prognosis

**Figure 3:**
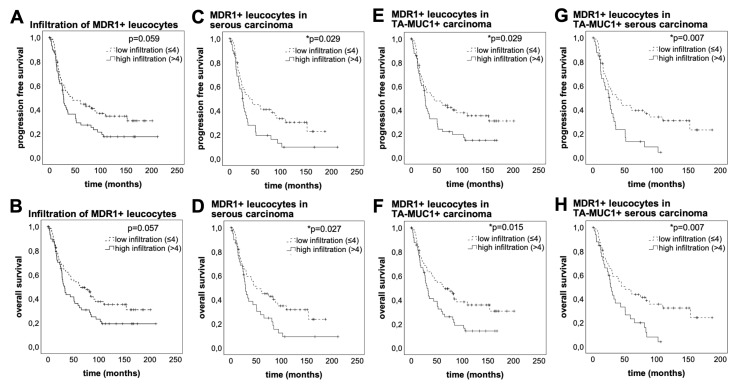
A high MDR1+ leucocyte infiltrate is associated with poor prognosis especially in patients whose carcinoma shows serous histology and TA-MUC1 expression. The Kaplan-Meier estimates show that high MDR1+ leucocyte infiltration (>4/field of view, 25× lens) leads to decreased PFS (**A**, *p* = 0.059, n = 126) and OS (**A**, *p* = 0.057, n = 126), although not statistically significant. Late separation of the curves suggests long time effects mediated by the MDR1+ leucocyte infiltrate. In serous subtype these effects lead to significantly impaired PFS (*p* = 0.029, n = 91) and OS (*p* = 0.027, n = 91). (**E**–**H**) show combined survival analysis of a high MDR1+ leucocyte infiltrate and TA-MUC1. PFS (E, *p* = 0.029, n = 110) and OS (F, *p* = 0.015, n = 110) of patients with high MDR1+ leucocyte infiltration is significantly decreased in TA-MUC1+ cases (IRS > 0), which are even worse when the carcinoma shows also serous histology (G, PFS, *p* = 0.007, n = 81; H, OS *p* = 0.007, n = 81). Censoring events have been marked in the graphs (+).

At the time of surgery, the median age of patients studied was 58.7 years (interquartile range (IQR) = 16.4) with a total range of 20.7–88.0 years. Median overall survival time after surgery was 33.8 months (IQR = 78.3) with a total range of 0–230.5 months. Regarding the non-stratified patient cohort, these with a high MDR1+ leucocyte infiltrate indicated a trend of impaired progression free survival (PFS) (median PFS 27.36 vs. 44.76 months, *p* = 0.059, n = 126) and OS (median OS 32.52 vs. 63.36 months, *p* = 0.057, n = 126), although not statistically significant (Figure 3A,B). Late separation of the curves suggests long term effects mediated by the MDR1+ immune cell infiltrate. The phenomenon of a delayed impact of immunological processes on survival has been described before [25]. Excluding the early deaths, which can be attributed to therapeutic consequences and bad general condition, the negative effect of a high MDR1+ leucocyte infiltrate becomes apparent (median OS 61.32 vs. 153.48 months, *p* = 0.031, n = 82) (Supplementary file Figure S5). Especially in patients whose carcinoma showed serous histology PFS (median PFS 39.6 vs. 67.16 months, *p* = 0.029, n = 91) and OS (median OS 28.80 vs. 50.64 months, *p* = 0.027, n = 91) were significantly decreased in case of a high MDR1+ leucocyte infiltrate (Figure 3C,D). Thus, a high MDR1+ leucocyte infiltrate indicates worse prognosis in ovarian cancer, notably in serous subtype.

A tendency of reduced survival was also shown for patients with membranous expression of TA-MUC1 [14]. Staining of TA-MUC1 was successfully performed with Gatipotuzumab, a therapeutic antibody which showed safety and tolerability but no clinical benefit in clinical trials so far [26,27]. 134 cases (85.9%) showed a positive TA-MUC1 expression with median IRS = 8 (total range 0–12) (Supplementary file Figure S6). As TA-MUC1 is known to be a tumor-specific immunogenic epitope [28], we hypothesized a possible relation to tumoral leucocyte infiltration. Strong positive correlation of TA-MUC1 expression of the tumor and MDR1+ leucocyte infiltration supported this assumption. Therefore, the prognostic relevance of the MDR1+ immune cell infiltrate in TA-MUC1+ patients (IRS > 0) was analyzed. Indeed, the PFS (median PFS 27.00 vs. 44.76 months, *p* = 0.029, n = 110) and OS (median OS 30.60 vs. 63.36 months, *p* = 0.015, n = 110) of TA-MUC1+ patients was significantly decreased in case their tumors showed a high MDR1+ immune cell infiltrate (Figure 3E,F). Moreover, combined survival analysis (Figure 3G,H) showed the worst prognosis for patients with TA-MUC1+ serous carcinoma in case of a high MDR1+ immune cell infiltrate (median PFS 25.80 vs. 39.60 months, *p* = 0.007, n = 81; median OS 27.84 vs. 50.64 months, *p* = 0.007, n = 81).

In order to asses independency of prognostic factors multivariate Cox regression analysis was conducted. With this method FIGO and infiltration with MDR1+ leucocytes turned out to be independent prognostic factors for OS and PFS (Table 3).

### 3.4. Identification of M2 Macrophages as Main Part of the Immune Cell Infiltrate

**Figure 4:**
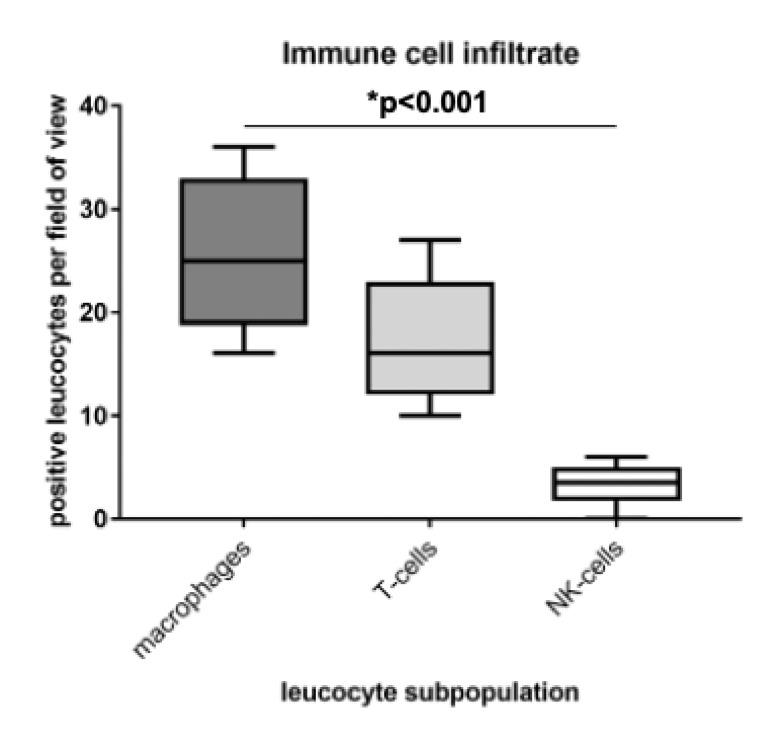
Macrophages were identified as main part of the immune cell infiltrate. The immune cell infiltrate was quantified by counting positive cells per field of view (20× lens; n = 12) in immunofluorescence double staining. Most infiltrating cells were CD68 positive macrophages, followed by CD3 positive T-cells. Just a few CD56 positive NK-cells were detected.

The identification and characterization of the MDR1+ immune cell infiltrate was conducted by immunofluorescence double staining on representative serous carcinoma samples. Firstly, the immune cell subpopulations were quantified by counting CD68, CD3 and CD56 positive cells per field of view (20× lens, n = 12 each). Most infiltrating cells were CD68 positive macrophages, followed by CD3 positive T-cells. Just a few CD56 positive NK-cells were detected (Figure 4).

**Figure 5:**
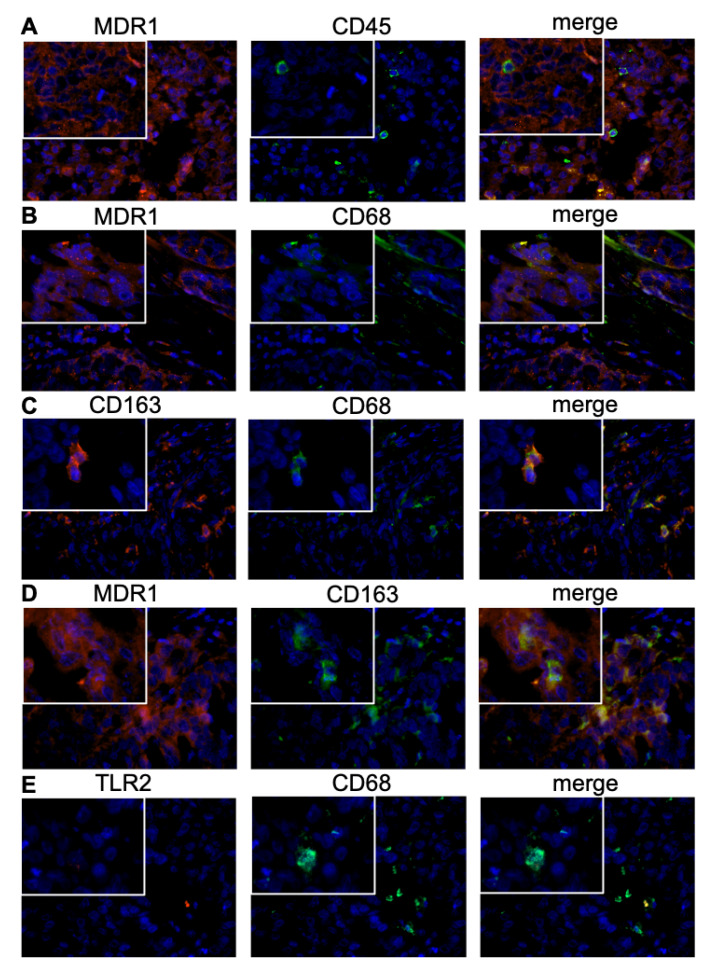
Characterization of the immune cell subpopulation by immunofluorescence double staining. M2 macrophages were identified as main part of the MDR1+ leucocyte infiltrate, expressing besides MDR1 the M2 marker CD163 (**D**) as well as the pan-macrophage marker CD68 (**B**). The stained tissue slices of serous ovarian cancer tissue were analyzed in 40× and 63× (inserts) magnification. For most CD45 positive immune cells (green) a co-localization with MDR1 (red) was observed (**A**); co-expression of MDR1 (red) and CD68 (green) (**B**); co-expression of CD163 (red) and CD68 (green) (**C**); co-expression of MDR1 (red) and CD163 (green) (**D**); no co-expression of TLR2 (red) and CD68 (green) was detected (**E**). Cell nuclei were marked by DAPI (blue) staining.

Subsequently, the MDR1 expressing leucocyte subpopulation was analyzed. For most CD45 positive immune cells a co-localization with MDR1 was observed (Figure 5A). M2 macrophages were identified as main part of the MDR1+ infiltrate, expressing besides MDR1 also the M2 marker CD163 and the pan-macrophage marker CD68 (Figure 5B–D). In contrast, no co-expression of TLR-2, accepted as M1 macrophage marker, and MDR1 was detected (Figure 5E). 96.3% of M2 macrophages were MDR1 positive (Figure 5D), whereas just a few MDR1 positive T-cells (Supplementary file Figure S7F) and no MDR1 positive NK-cells (Supplementary file Figure S7E) were observed.

## 4. Discussion

In this study, MDR1 expression has been detected on both ovarian cancer cells and infiltrating immune cells of all histological subtypes. Elevated MDR1 expression is considered to be one of the main mechanisms of chemoresistance in many cancer types [5]. In ovarian cancer enhanced MDR1 expression leads to reduced paclitaxel response and to worse prognosis [7]. In this study, we demonstrate that a high infiltrate of MDR1+ leucocytes to the tumor and the peritumoral stroma has an additional negative impact on prognosis. Univariate analysis of OS indicates long-term effects on survival mediated by MDR1+ infiltrating leucocytes, particularly in serous subtype. A delayed impact of immunological processes on survival has been described before [25]. Similar findings for PFS suggest an influence of MDR1+ immune cells on the development of chemoresistance. Multivariate analysis proved independency of a high MDR1+ leucocyte infiltrate as prognostic factor for OS and PFS. However, the residual disease after primary surgery was not included in the Cox regression model due to insufficient data, which limits the analysis. Regarding the TA-MUC1+ subgroup, the effects of a high MDR1+ leucocyte infiltrate become even more evident. As TA-MUC1 is known to be a tumor-specific immunogenic epitope [28], we hypothesized a possible relation to tumoral leucocyte infiltration. Strong positive correlations with the immunogenic epitopes HER2 and TA-MUC1 support this assumption, while no associations with clinical characteristics were found.

In cancer biology, the role of an immune cell infiltrate has different functional aspects. Depending on the population and the activation status, it can either inhibit or promote tumor progression [29]. Especially Tregs and M2 macrophages, which are often found in the TME, mediate protumoral effects [9,10]. In the present analysis, the subpopulation of M2 macrophages was identified as main part of the MDR1+ immune cell infiltrate by immunofluorescence co-localization. In accordance with previous studies [30,31], we prove that MDR1+ M2 macrophages lead to poor prognosis. Additionally, we show that M2 macrophages in ovarian cancer express MDR1 to a high extend and percentage, which may account for the poor prognosis due to an impact on drug resistance. Concordantly, Cory et al. described alterations in MDR1 expression and function between the macrophage subsets with M2 polarized macrophages showing a higher expression and function [32]. Consequently, the immunosuppressive M2 phenotype achieves resistance to chemotherapies and survival advantages compared to M1 macrophages by MDR1 as described for tumor cells. These findings suggest that M2-type tumor-associated macrophages (TAMs) may play an important role in drug resistance mechanisms in the TME and provides a valuable basis for further investigations concerning the functional role of MDR1+ M2 macrophages in ovarian cancer or other tumor entities, which might have therapeutic consequences. Paclitaxel, the standard combination partner for platinum-based chemotherapy in ovarian cancer, not only induces cell cycle arrest via microtubule stabilization but also promotes anti-tumoral immunity. Former studies showed that treatment with paclitaxel altered the M2-like signature toward an M1-like profile reducing tumor growth [33]. As paclitaxel is a substrate of MDR1, also this effect may be limited in MDR1+ M2 macrophages. Therefore, a combination of paclitaxel treatment with MDR1 inhibitors could be a promising perspective for future therapeutic approaches.

The interaction of cancer and immune cells is regulated by soluble factors and surface proteins [9]. During malignant transformation cells change their expression pattern and immunogenic tumor-associated antigens (TAAs) emerge. Interestingly, we found strong correlations between the MDR1+ leucocyte infiltrate and the well-known TAAs HER2 and TA-MUC1. Both epitopes have been demonstrated to induce a humoral autoantibody response [28] and seem to be immunogenic. In breast cancer for example, patients with anti-TA-MUC1 autoantibodies had a significant survival benefit [34], which indicates the activation of an anti-tumoral immune defense. To enhance the natural immunoactivation, therapeutic antibodies against those cancer specific targets have been developed.

Despite immunoactivation, alterations in glycosylation patterns contribute to the immune evasion of cancers. In contrast to normal epithelial cells, glycosylations of mucins on cancer cells are short and sialylated [35]. It was reported that TAMs express high levels of lectins, which mediate the mucin ligation, in ovarian carcinoma [36]. More specifically, binding of sialylated TA-MUC1 to Siglec-9 on primary macrophages induces polarization to the M2 phenotype confirmed by the upregulation of M2 markers like CD163 [35]. Therefore, it is in accordance that overexpression of MUC1 in transgenic mice increased the recruitment of M2-type macrophages and created a proinflammatory environment, which promoted cancer development [37]. Inferring from this evidence TA-MUC1 seems to be a responsible factor for the high occurrence of M2 macrophages detected in the ovarian cancer samples. Furthermore, recent studies show a link between MUC1 and chemoresistance, impeding intracellular drug uptake and activating a cell signaling cascade leading to an attenuation of oxidative stress [38,39].

Taken together, we observed that a high infiltrate of MDR1+ immune cells is an independent prognostic factor for long term survival of ovarian cancer patients. In particular, PFS and OS are significantly decreased in case of a high MDR1+ leucocyte infiltrate in patients whose tumors show serous histology and TA-MUC1 expression. By immunofluorescence co-localization we identified M2 macrophages as main part of the immune cell infiltrate and show that MDR1 is highly expressed in M2 macrophages, giving them a survival advantage in cytotoxic treatment. TA-MUC1 and an immunosuppressive TME mediated by M2-type macrophages seem to be relevant causes for impaired prognosis and chemoresistance. To confirm the hypothesis that MDR1+ M2 macrophages play a crucial role in the development of chemoresistence, further functional analysis are necessary. Paclitaxel in combination with MDR1 inhibitors could be an interesting therapeutic approach to repolarize macrophages and promote anti-tumoral immunity which warrants further investigation. Thus, this study provides a valuable basis for further investigations concerning the functional role of MDR1+ M2 macrophages in ovarian cancer or other tumor entities, which might have therapeutic consequences.

## Figures and Tables

**Table 1:** Clinicopathologic characteristics of the ovarian cancer patients.

Clinicopathologic Parameters	n	Percentage (%)
*Histology*		
Serous	110	70.5
Clear cell	12	7.7
Endometrioid	21	13.5
Mucinous	13	8.3
*Primary tumor expansion*		
TX	1	0.6
T1	40	25.6
T2	18	11.5
T3	97	62.3
*Nodal status*		
pNX	61	39.1
pN0	43	27.6
pN1	52	33.3
*Distant Metastasis*		
pMX	147	94.2
pM0	3	1.9
pM1	6	3.8
*Grading Serous*		
Low	24	21.8
High	80	72.7
*Grading Endometrioid*		
G1	6	28.6
G2	5	23.8
G3	8	38.1
*Grading Mucinous*		
G1	6	46.2
G2	6	46.2
G3	0	0
*Grading Clear cell*		
G3	12	100.0
*FIGO*		
I	35	22.4
II	10	6.4
III	103	66.0
IV	3	1.9
*Residual disease after primary surgery*		
unknown	143	91.7
complete cytoreduction	11	7.1
incomplete cytoreduction	2	1.3
*Age*		
≤60 years	83	53.2
>60 years	73	46.8

**Table 2:** Correlation analysis between MDR1+ leucocyte infiltrate and other pathological markers.

Staining	MDR1+ Leucocytes	HER2	TA-MUC1
*MDR1+ leucocytes*			
Cc	1.000	0.258	0.202
p	-	0.005 *	0.022 *
n	139	119	127
*HER2*			
Cc	0.258	1.000	0.008
p	0.005 *	-	0.924
n	119	152	139
*TA-MUC1*			
Cc	0.202	0.008	1.000
p	0.022 *	0.924	-
n	127	139	143

IRS of HER2 and TA-MUC1 were correlated to each other and to the number of infiltrating MDR1+ leucocytes per field of view using Spearman’s correlation analysis. Significant correlations are indicated by asterisks (*: *p*< 0.05). Cc: correlation coefficient; p: two-tailed significance; n: number of patients.

**Table 3:** FIGO and a high MDR1+ leucocyte infiltration are independent prognostic factors for OS and PFS.

Covariate		*p*	Hazard Ratio (95% CI)
Subtype (serous vs. mucinous, endometrioid and clear cell)	**OS**	0.680	1.145 (0.601–2.184)
	**PFS**	0.365	1.341 (0.711–2.526)
FIGO I-IV (continous)	**OS**	<0.001 *	2.471 (1.633-3.740)
	**PFS**	<0.001 *	2.433 (1.622–3.679)
MDR1+ leucocyte infiltration high (>4) vs. low (≤4)	**OS**	0.013 *	1.816 (1.136–2.904)
	**PFS**	0.008 *	1.881 (1.176–3.009)

A multivariate Cox regression model was established to investigate independency of prognostic factors. Significant independent factors are indicated by asterisks (*: *p* < 0.05). CI: confidence interval; OS: overall survival; PFS: progression free survival.

## Supplementary Materials

The following are available online at https://www.mdpi.com/2073-4409/9/5/1224/s1, Figure S1: Positive and negative system control for MDR1 staining; Figure S2: MDR1 immunostaining in healthy ovary; Table S1: Antibodies used for immunouorescence co-staining; Figure S3: MDR1 immunostaining of ovarian cancer cells; Figure S4: MDR1 positive leucocyte infiltrate was detected by immunohistochemistry in all subtypes; Table S2: Correlation analysis between MDR1+ leucocyte infiltration and clinicopathological data for the whole collective; Figure S5: The MDR1+ leucocyte infiltrate mediates long term effects; Figure S6: TA-MUC1 (Gatipotuzumab) staining of ovarian cancer tissue of the same individuals shown in Figure 2; Figure S7: Identication and characterization of the immune cell subpopulation by immune uorescence double staining.

## Abbreviations

The following abbreviations are used in this manuscript:

ADP	adenosine diphosphate
ATP	adenosine triphosphate
CC	correlation coefficient
CD	cluster of differentiation
CI	confidence interval
DAPI	4’,6-diamidino-2-phenylindole
EOC	epithelial ovarian cancer
FFPE	formalin-fixated and paraffin-embedded
FIGO	Fédération Internationale de Gynécologie et d’Obstétrique
HER2	human epidermal growth factor receptor 2
HR	hazard ratio
IQR	interquartile range
IRS	immunoreactive score
MDR	multidrug resistance
MDR1	multidrug resistance protein 1, p-glycoprotein
NK-cells	natural killer cells
OS	overall survival
PBS	phosphate buffered saline
PFS	progression free survival
ROC	receiver operating characteristic
TA-MUC1	tumor-associated mucin 1
TAAs	tumor-associated antigens
TAMs	tumor-associated macrophages
TME	tumor microenvironment
WHO	World Health Organization
