# Supplementary material for: M2 Macrophages Infiltrating Epithelial Ovarian Cancer Express MDR1: A Feature That May Account for the Poor Prognosis
Source: Cells. 2020 May 15;9(5):1224. doi: 10.3390/cells9051224 (PMC7290705; doi:10.3390/cells9051224)
Supplement: Supplementary file 1 [file cells-09-01224-s001.pdf]

Supplementary file to

M2 Macrophages infiltrating epithelial ovarian cancer express MDR1: A feature that may account for the poor prognosis

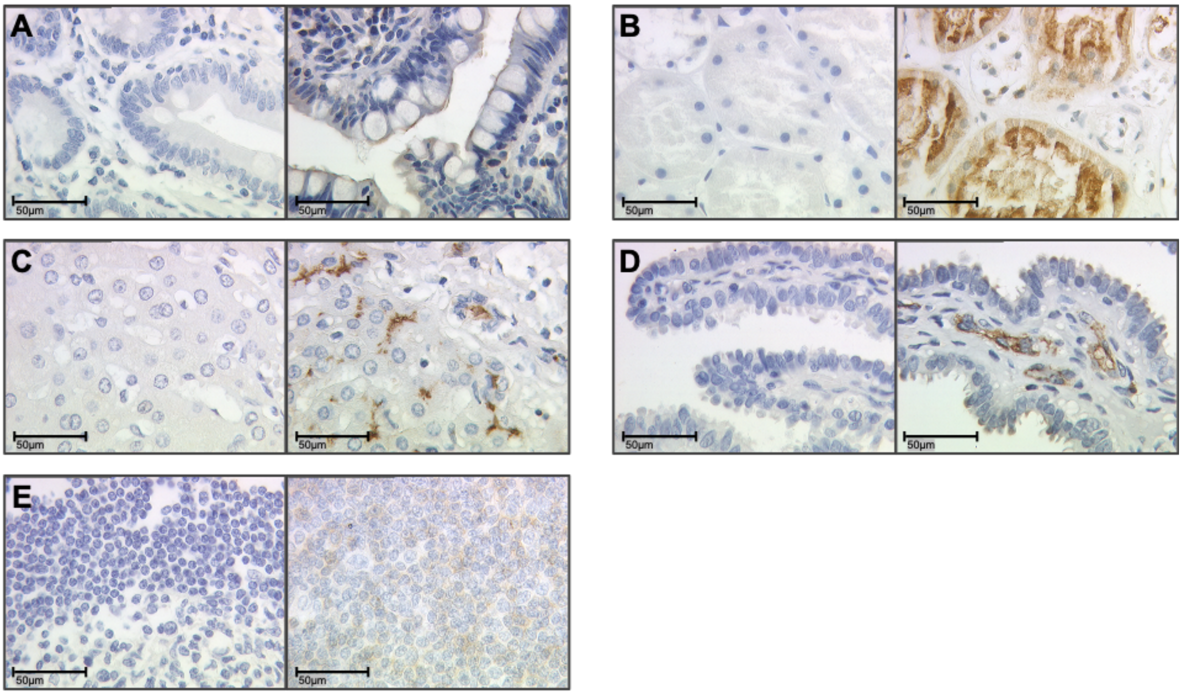

Figure S1 Negative (left) and positive (right) system control for MDR1 staining in tissue form human small intestine (A), kidney (B), liver (C), fallopian tube (D) and tonsil (E). A-E are shown in 40x (scale bar=50 µm) magnification.

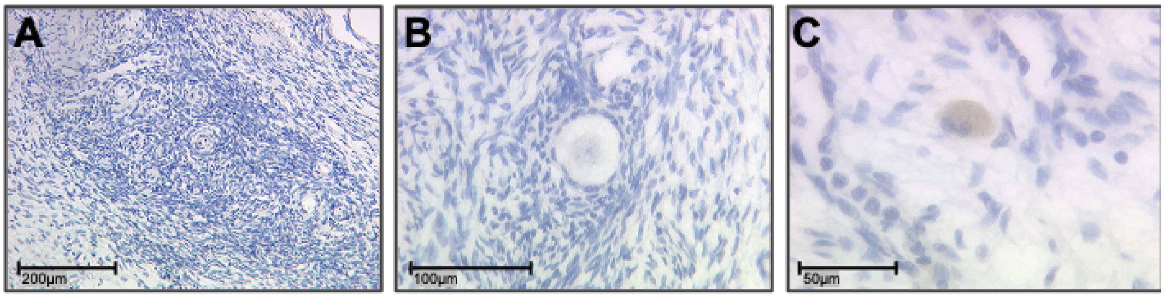

Figure S2 MDR1 immunostaining in healthy ovary. In contrast to the area of the tumor, healthy ovarian tissue (nighter spinocellular connective tissue nor follicles) was not positive for MDR1. Only sporadic MDR1+ infiltrating immune cells were found (C), but in lower density compared to EOC. A 10x (scale bar=200 µm), B 25x (scale bar=100 µm), C 40x (scale bar=50 µm) magnification.

Table S3 Antibodies used for immunofluorescence co-staining.

**Antibody**	**Dilution**	**Manufacturer**
Primary antibodies		
MDR1, monoclonal rabbit IgG	1:100	Abcam, Cambridge, UK
CD68, monoclonal mouse IgG1	1:1000	Sigma Aldrich, St. Louis, MO, USA
CD163, polyclonal rabbit IgG	1:2000	Sigma Aldrich, St. Louis, MO, USA
CD163, monoclonal mouse IgG1	1:800	Abcam, Cambridge, UK
CD3, monoclonal mouse IgG1	1:75	Dako, Glostrup, Denmark
CD45, monoclonal mouse IgG1	1:200	Dako, Glostrup, Denmark
CD56, monoclonal mouse IgG1	1:100	Serotec, Puchheim, Germany
TLR2, monoclonal mouse IgG1	1:800	Novusbio, Centennial, CO, USA
TLR2, monoclonal rabbit IgG	1:100	Abcam, Cambridge, UK
Secondary antibodies		
Cy3-conjugated goat-anti-rabbit IgG	1:500	Dianova, Hamburg, Germany
Alexa Fluor 488-conjugated goat-anti-mouse IgG	1:100	Dianova, Hamburg, Germany

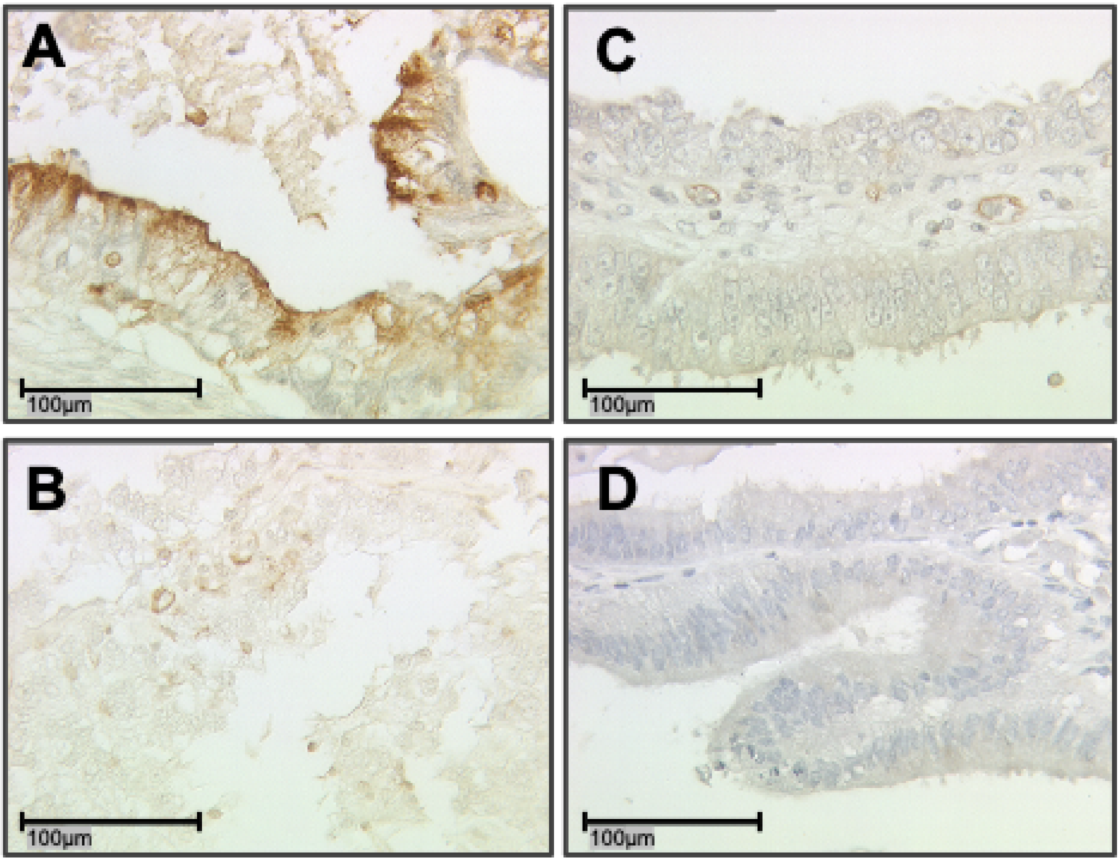

Figure S4 MDR1 immunostaining of ovarian cancer cells. Membranous expression of MDR1 on ovarian cancer cells differs between the subtypes with mucinous (A, IRS=6) and clear cell (B, IRS=4) showing a higher expression than serous (C, IRS=3) and endometrioid (D, IRS=2). A-D are shown in 25x magnification (scale bar=100 µm).

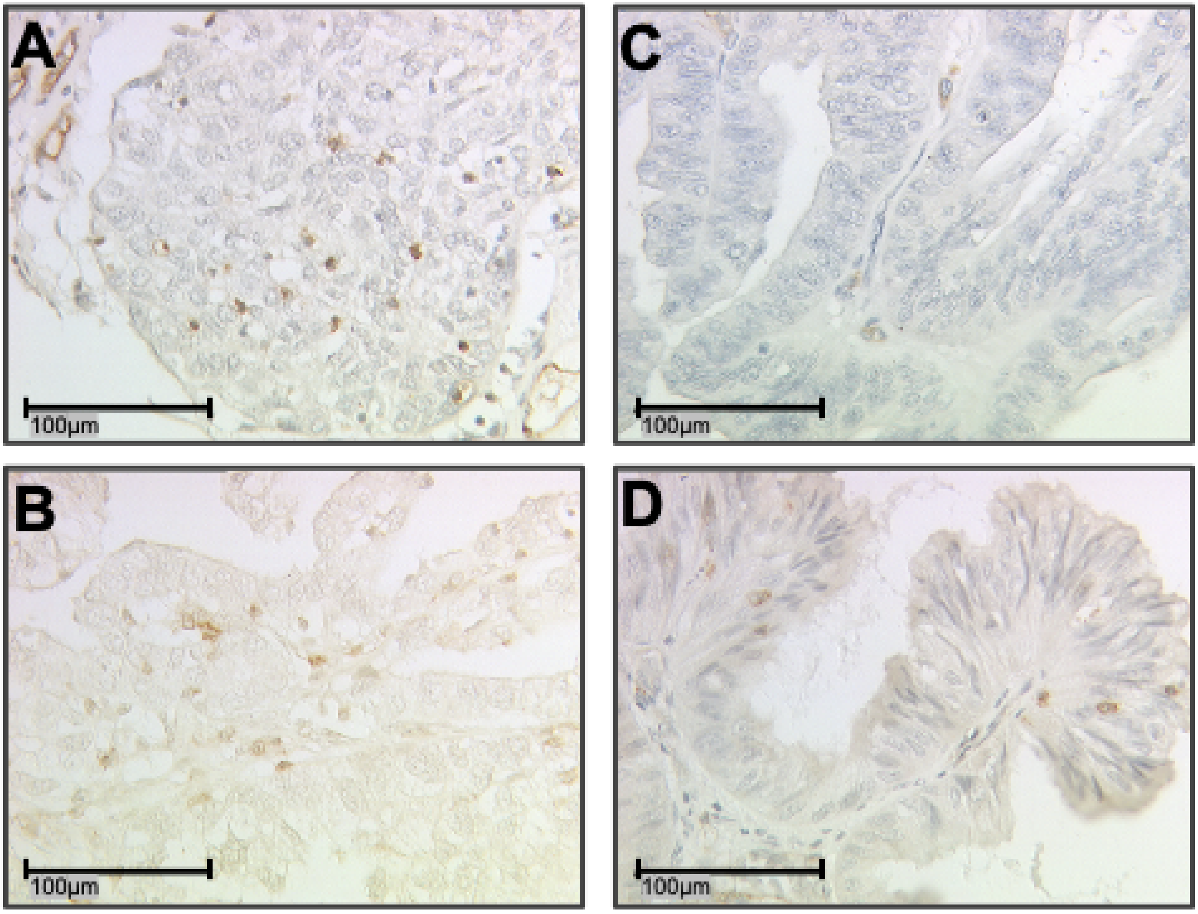

Figure S5 MDR1 positive leucocyte infiltrate was detected by immunohistochemistry in all subtypes: serous (A), clear cell (B), endometrioid (C) and mucinous carcinoma (D). A-D are shown in 25x magnification (scale bar=100 µm).

Table S6 Correlation analysis between MDR1+ leucocyte infiltration and clinicopathological data for the whole collective.

	Age	Subtype	FIGO	pT	pN	Grading clear cell, mucinous, endometrioid	Grading serous	MDR1+ leucocyte infiltration
Age								
Cc	1	-0.127	0.16	0.203*	-0.014	-0.091	0.299**	0.055
p	-	0.113	0.05	0.011	0.894	0.268	0	0.537
n	156	156	151	155	95	151	156	127
Subtype								
Cc	-0.127	1	-0.404**	-0.374**	-0.366**	0.945**	-0.652**	0.031
p	0.113	-	0	0	0	0	0	0.729
n	156	156	151	155	95	151	156	127
FIGO								
Cc	0.16	-0.404**	1	0.841**	0.764**	-0.334**	0.435**	0.03
p	0.05	0	-	0	0	0	0	0.744
n	151	151	151	151	92	148	151	123
pT								
Cc	0.203*	-0.374**	0.841**	1	0.587**	-0.318**	0.518**	0.084
p	0.011	0	0	-	0	0	0	0.349
n	155	155	151	155	95	151	155	126
pN								
Cc	-0.014	-0.366**	0.764**	0.587**	1	-0.225*	0.251*	0.053
p	0.894	0	0	0	-	0.031	0.014	0.641
n	95	95	92	95	95	92	95	81
Grading clear cell, mucinous, endometrioid								
Cc	-0.091	0.945**	-0.334**	-0.318**	-0.225*	1	-0.629**	0.05
p	0.268	0	0	0	0.031	-	0	0.584
n	151	151	148	151	92	151	151	123
Grading serous								
Cc	0.299**	-0.652**	0.435**	0.518**	0.251*	-0.629**	1	0.071
p	0	0	0	0	0.014	0	-	0.424
n	156	156	151	155	95	151	156	127
MDR1+ leucocyte infiltration								
Cc	0.055	0.031	0.03	0.084	0.053	0.05	0.071	1
n	0.537	0.729	0.744	0.349	0.641	0.584	0.424	-
n	127	127	123	126	81	123	127	127

Clinicopathologic data and a MDR1+ leucocyte infiltrate were correlated to each other using Spearman's correlation analysis. Significant correlations are indicated by asterisks (*: p<0.05; **: p<0.01). Cc: correlation coefficient, p: two-tailed significance, n: number of patients.

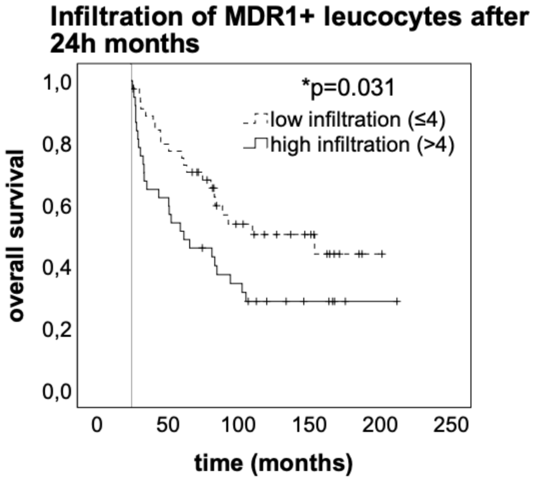

Figure S7 The MDR1+ leucocyte infiltrate mediates long term effects. Excluding the early deaths, which can be attributed to therapeutic consequences and bad general condition, the prognostic negative effect of a high MDR1+ leucocyte infiltrate becomes apparent (p=0.031, n=82).

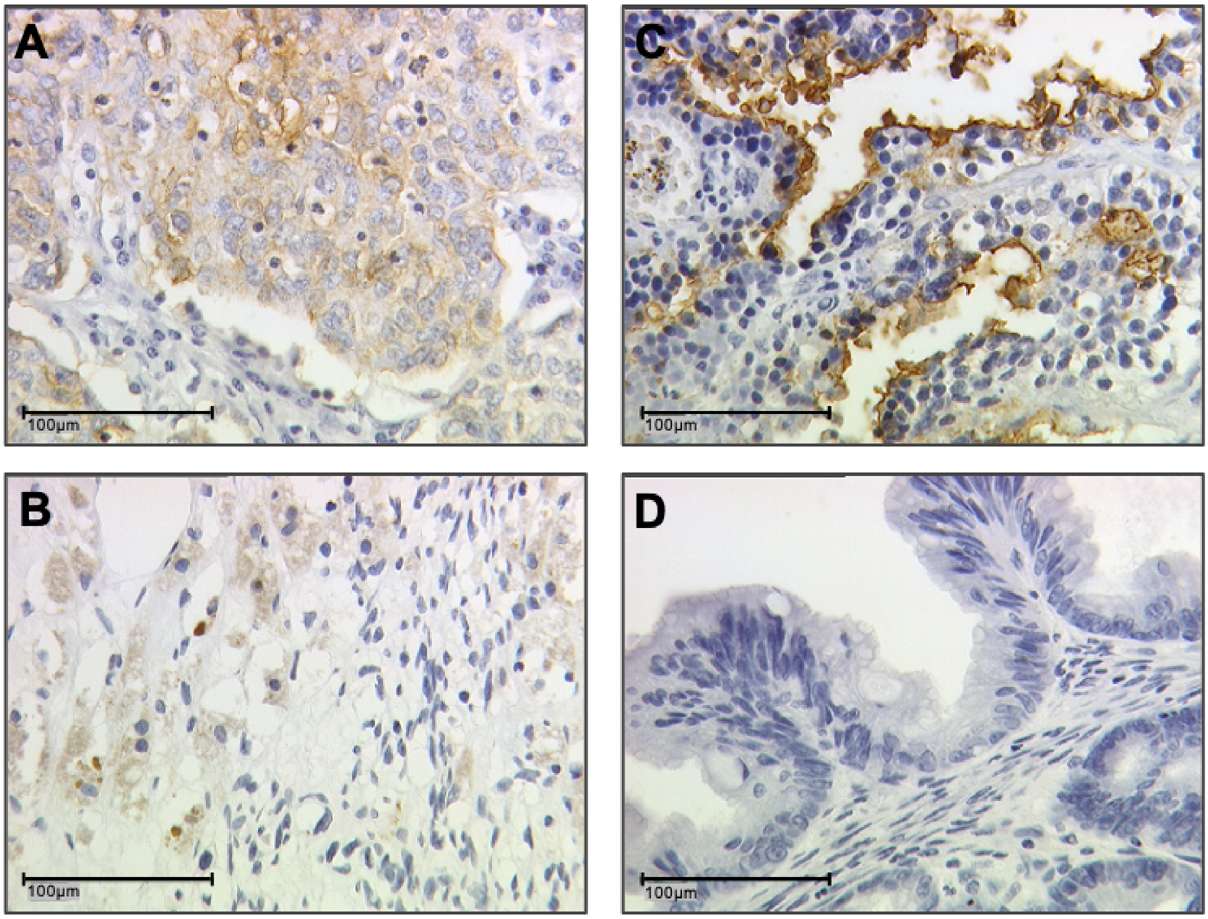

Figure S8 TA-MUC1 (Gatipotuzumab) staining of ovarian cancer tissue of the same individuals shown in Figure 2. A serous IRS=8; B clear cell IRS=9; C endometrioid IRS=10; D mucinous IRS=1. A-D 25x (inserts, scale bar=100 µm) magnification. Correlation analysis showed a significant positive correlation between TA-MUC1 expression of the tumor and MDR1+ leucocyte infiltration ($p=0.022$).

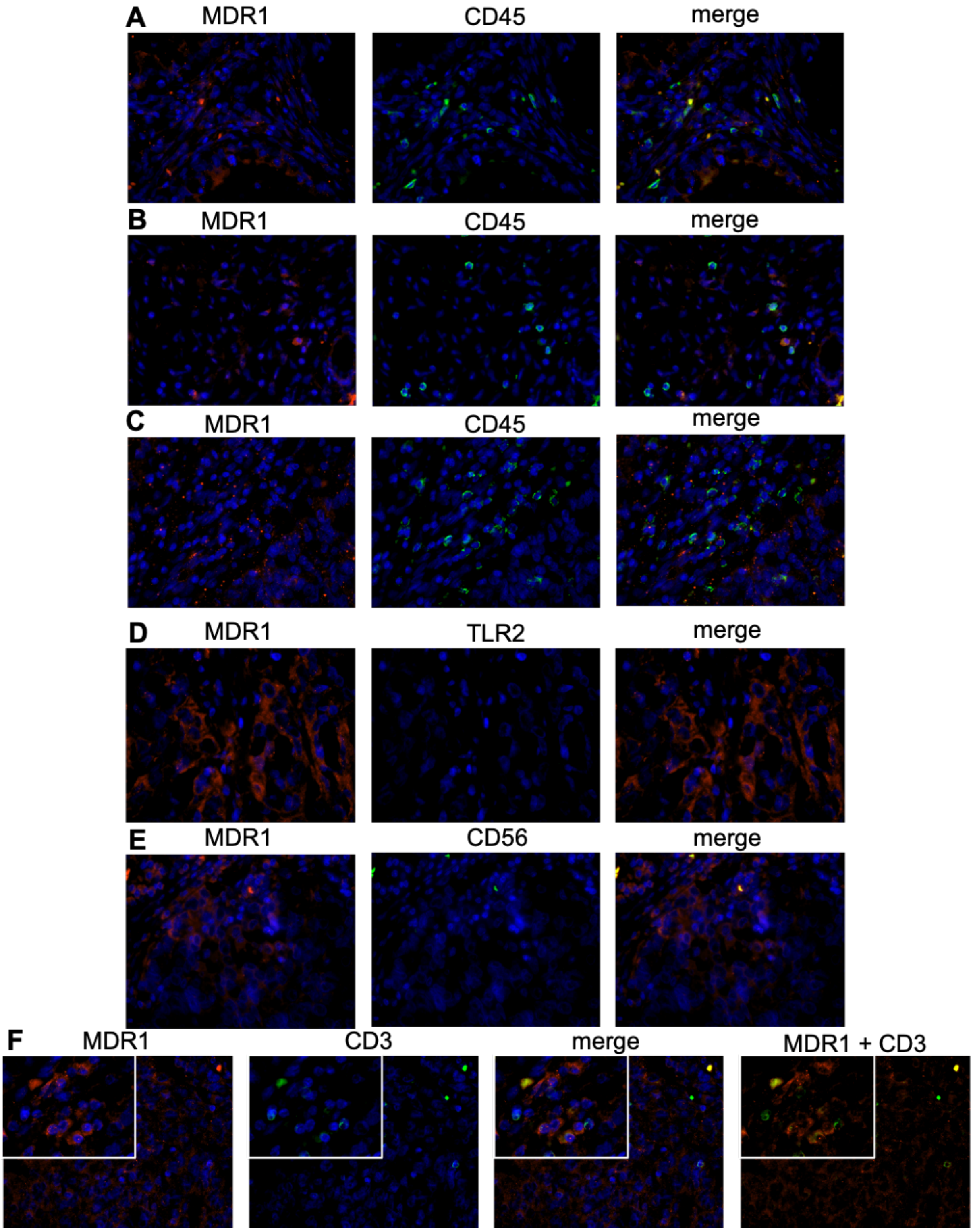

Figure S9 Identification and characterization of the immune cell subpopulation by immunofluorescence double staining. For most CD45 positive immune cells (green) a co-localization with MDR1 (red) was observed (A mucinous, B clear cell, D endometrioid carcinoma). Due to negative co-expression of MDR1 (red) and TLR2 (green) (D) and MDR1 (red) and CD56 (green) (E) M1 macrophages and NK-cells can be excluded. In some T-cells co-expression of MDR1 (red) and CD3 (green) was found but in a fewer amount than in macrophages (F). Cell nuclei were marked by DAPI (blue) staining. The pictures were analyzed in 40x respectively 63x (inserts) magnification.
